# Contribution of SPI-1 bistability to *Salmonella enterica* cooperative virulence: insights from single cell analysis

**DOI:** 10.1038/s41598-018-33137-z

**Published:** 2018-10-05

**Authors:** María Antonia Sánchez-Romero, Josep Casadesús

**Affiliations:** Departamento de Genética, Facultad de Biología, Universidad de Sevilla, Apartado 1095, 41080 Sevilla, Spain

## Abstract

*Salmonella enterica* pathogenicity island 1 (SPI-1) is a gene cluster that encodes a type III secretion system and effectors involved in epithelial cell invasion. SPI-1 undergoes bistable expression, with concomitant formation of SPI-1^**ON**^ and SPI-1^**OFF**^ lineages. This study describes single cell analysis of SP1-1 bistability and epithelial cell invasion, and reports the unsuspected observation that optimal invasion of epithelial cells requires the presence of both SPI-1^**ON**^ and SPI-1^**OFF**^ subpopulations. The contribution of SPI-1^**OFF**^ cells to optimal invasion may rely on their ability to invade epithelial cells if a SPI-1^**ON**^ subpopulation is present. In fact, *Salmonella* SPI-1 mutants are also able to invade epithelial cells in the presence of SPI-1^**ON**^ *Salmonellae*, a phenomenon described in the 1990’s by Galán and co-workers. Invasion by SPI-1^**OFF**^ cells does not seem to involve a diffusible factor. A small number of SPI-1^**ON**^ cells is sufficient to endow the bacterial population with invasion capacity, a feature that may permit host colonization regardless of the bottlenecks encountered by *Salmonella* populations inside animals.

## Introduction

The notion that isogenic bacterial populations contain cells with different phenotypes is widely accepted in today’s microbiology^1,2^. Certain cell-to-cell phenotypic differences are merely a consequence of noisy gene expression^3,4^; in other cases, however, phenotypic heterogeneity is a programmed event under genetic or epigenetic control^5–7^. In such cases, the bacterial population splits into subpopulations showing distinct phenotypes, a phenomenon known as multistability^8^. Most examples of multistability validated by experimental analysis involve two phenotypic states only (bistability)^6,9^. When reversion of the bistable states is a programmed event, the phenomenon is known as phase variation^6,10,11^.

Formation of bacterial subpopulations can provide two main types of benefits, division of labour and preadaptation to environmental change (“bet hedging”)^7,12^. Division of labour has adaptive value in a constant environment, and the payoff of each subpopulation depends on its specific contribution. In bet hedging, each subpopulation is adapted to thrive under different conditions and the benefit for the entire population shows off only in a fluctuating environment^13^. Because each bet hedging subpopulation is well adapted to a given environment only, subpopulations pay a toll under unfavourable circumstances, and maintenance of bistability may be viewed as a tradeoff^13^. For instance, phase variation of the *Salmonella enterica opvAB* operon generates a bacterial subpopulation that is resistant to phages at the expense of virulence attenuation^14^. Another example of tradeoff may be found in phase-variable glycosyltransferase (*gtr*) operons^15^, whose products are crucial for intestinal persistence and faecal shedding of *Salmonella* but reduce invasion of both epithelial cells and macrophages^16^. In both examples, programmed reversion of the bistable states regenerates heterogeneity and sustains the tradeoff.

A conundrum regarding phenotypic heterogeneity concerns its evolution: because subpopulation formation may benefit the entire population rather than the individual subpopulations, its evolutionary emergence may require group selection. In classical darwinism, the unit of selection is the individual rather than the population, and group selection is considered a weak evolutionary force^17,18^. This classical view is however countered by game theory models indicating that phenotypic heterogeneity can have selective value^19–21^.

A paradigm of programmed bistability is found in pathogenicity island 1 (SPI-1) of *Salmonella enterica* serovar Typhimurium^22–24^. SPI-1 is a ~40 kb gene cluster that encodes a type III secretion system (T3SS) and T3SS-secreted effectors involved in invasion of epithelial cells^25–27^. SPI-1 shows bistable expression in the mouse gut and under laboratory conditions that mimic the intestinal environment: building of the T3SS occurs in a subpopulation of bacterial cells only^28,29^. The SPI-1^ON^ phenotype is heritable, and persists for several generations if the bacterial population is shifted to environments where SPI-1 is not induced^30^. Unlike other bistable systems which are controlled by relatively simple feedback loops^9^, SPI-1-expression is subjected to multiple, entangled transcriptional and postranscriptional controls^31–35^, and the mechanisms that control bistability remain under investigation.

Wolf-Dietrich Hardt and co-workers have combined modelling and experimental analysis to ponder the adaptive value of SPI-1 bistability, and have unveiled specific payoffs and tradeoffs of subpopulation formation. The SPI-1^ON^ subpopulation synthesizes the machinery for epithelial cell invasion and the SPI-1^OFF^ subpopulation does not; however, SPI-1^OFF^ cells benefit from inflammation triggered by the T3SS. As a consequence of inflammation, reactive oxygen species produced by phagocytes oxidize endogenous sulfur compounds to produce tetrathionate, and respiration of tetrathionate confers a growth advantage to *S*. *enterica* serovar Typhimurium over competing intestinal microbes^36,37^. As a payoff for their invasion capacity, the SPI-1^ON^ subpopulation shows retarded growth, which may reflect the burden of building the secretion apparatus and keeping it active^30^. However, as a compensation for slow growth, the SPI-1^ON^ subpopulation shows higher resistance to antibiotics^38^. SPI-1 bistability may thus be viewed as a division of labor during infection, but also as a bet hedging that preadapts the population to survive in the presence of antibiotics. Hardt and co-workers have also shown that the payoffs and tradeoffs of SPI-1 bistability fit in a model of “cooperative virulence”: fast growing SPI-1^OFF^ *Salmonella* cells can compete with avirulent mutants, thereby preventing a potential takeover of the population by *Salmonella* avirulent variants^39^. Increased resistance to antibiotics strengthens cooperative virulence^40^.

Cooperative virulence in the invasion of epithelial cells by *Salmonella enterica* may be seen as an example of Maynard Smith’s “evolutionarily stable strategy” in the microbial world. According to this classical notion of game theory, an evolutionary strategy becomes stable if natural selection alone is sufficient to prevent any alternative strategy that is initially rare^41^. In cooperative virulence, formation of SPI-1^OFF^ and SPI-1^ON^ subpopulations preserves *Salmonella* virulence in the entire population, preventing mutation as an alternative strategy^39^.

This study analyzes SP1-1 bistability by single cell analysis, an approach that permits assessment of subpopulation sizes, use of sorted subpopulations in invasion assays, and other tests not amenable to whole-culture microbiology. We report that optimal invasion of epithelial cells requires the presence of both SPI-1^ON^ and SPI-1^OFF^ subpopulations. However, *Salmonella* populations containing SPI-1^ON^ and SPI-1^OFF^ cells are invasive regardless of their proportions. In fact, a small number of SPI-1^ON^ cells appears to be sufficient for invasion of epithelial cells. Flexible division of labour may foster invasion regardless of the size of the SPI-1^ON^ and SPI-1^OFF^ subpopulations, thus conferring robustness to SPI-1 bistability. We also report the presence of both SPI-1^ON^ and SPI-1^OFF^ *Salmonellae* inside epithelial cells, a phenomenon previously known as “rescue”^42^. We hypothesize that rescue may strengthen cooperative virulence due to the ability of SPI-1^OFF^ *Salmonellae* to compete with non-invasive mutants in the intracellular environment.

## Results

### Pattern of SPI-1 expression along the growth cycle

Invasion of epithelial cells by *S. enterica* serovar Typhimurium requires the expression of genes encoded on pathogenicity island 1 (SPI-1) (reviewed in^43^). To monitor expression of SPI-1 at the single cell level, transcriptional green fluorescent protein (GFP) fusions were constructed downstream of three SPI-1 genes: *sipB*, which encodes an effector of  SPI-1 ; *prgH*, encoding a component of the T3SS apparatus; and *hilA*, a regulatory gene that encodes a transcriptional activator of SPI-1 operons.

**Figure 1:**
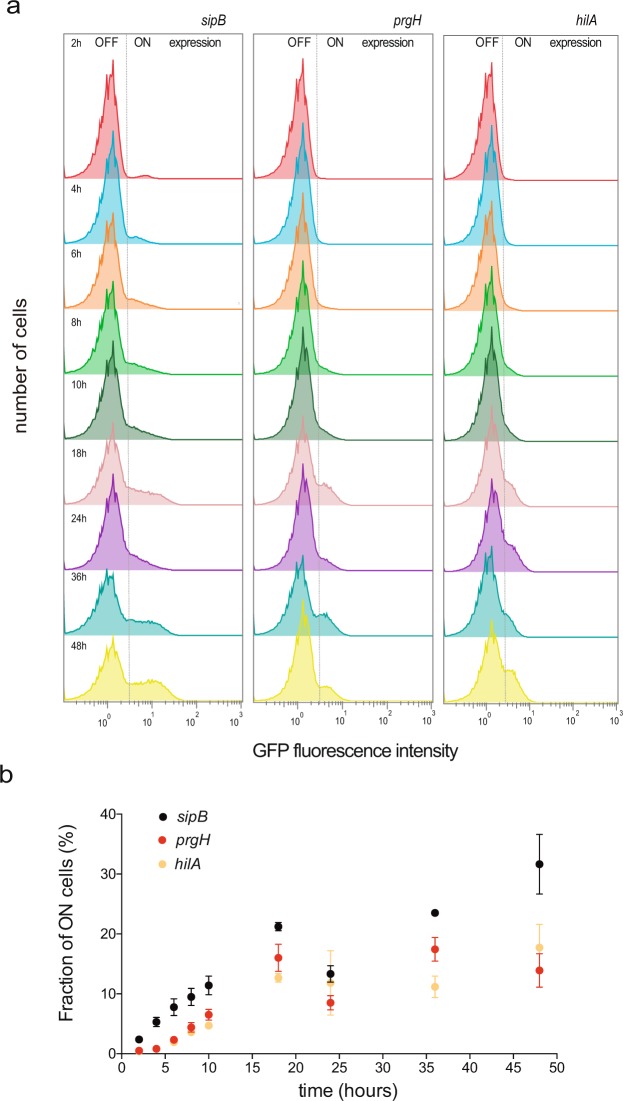
Flow cytometry analysis of the time course of SPI-1 expression during *Salmonella* growth. (**a**) GFP fluorescence intensity distribution in strains SV7884 (*sipB*::*GFP*), SV8348 (*prgH*::GFP), and SV6806 (*hilA*::GFP) grown in LB under oxygen-limited conditions. (**b**) Fraction of cells expressing three SPI-1 genes over time: *sipB::*GFP (black dots); *prgH*::GFP (red dots); and *hilA*::GFP (yellow dots).

**Figure 2:**
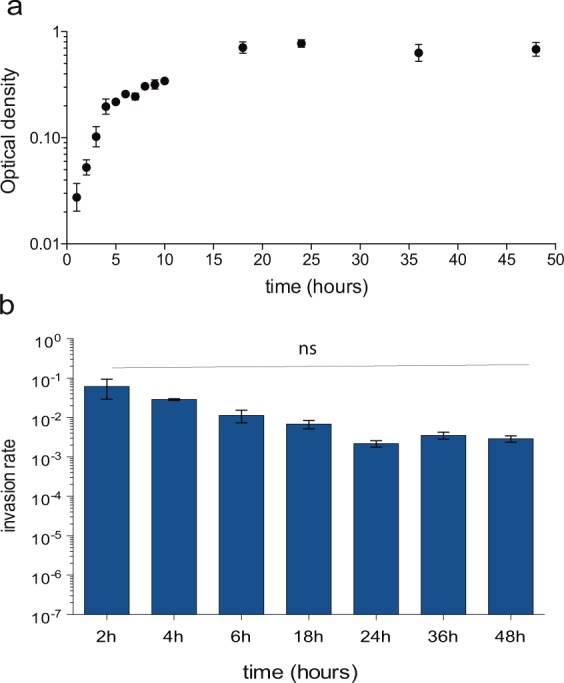
(**a**) Bacterial cell growth (optical density at 600 nm, black dots) over time. (**b**) Rates of epithelial cell invasion at different stages of the cell cycle. Averages and standard deviations from >6 independent experiments are shown. Statistical indications: ns, not significantly different.

The *sipB*::GFP, *prgH*::GFP, and *hilA*::GFP fusions were used to monitor the pattern of SPI-1 expression along the growth cycle using flow cytometry. Cultures were grown in LB under oxygen-limited conditions. The proportion of SPI-1^ON^ cells increased along the growth curve and the SPI-1^ON^ subpopulation attained its maximal size in stationary phase (Figs 1, 2a). The increase in the proportion of SPI-1^ON^ cells was confirmed by immunostaining of 3xFLAG-tagged SipB (Supplementary Fig. S1). These observations were merely confirmatory as the increase in SPI-1^ON^ cells along growth is a known trait of SPI-1 expression (Song *et al*., 2004; Sturm *et al*.^30^). However, the presence of SPI-1^ON^ cells at all stages of growth raised the possibility that a threshold might exist in the proportion of SPI-1^ON^ cells necessary to confer invasiveness. To address this issue, we measured the ability of an *S*. *enterica* culture to invade epithelial HeLa cells at different stages of bacterial growth. To our surprise, the invasion rate of the population remained more or less constant regardless of the proportion of SPI-1^ON^ cells (or decreased slightly as the number of SPI-1^ON^ cells increased) (Fig. 2).

Absence of a threshold had a paradoxical side as one might naïvely expect that higher proportions of SPI-1^ON^ cells would result in higher invasion rates. In fact, in many laboratories including ours, invasion assays are typically performed using late logarithmic or early stationary cultures, when SPI-1 bistability is conspicuous in the bacterial culture. However, our observations indicate that the invasion rate remains similar along the growth cycle as long as bistability is present in the bacterial population (Fig. 2; Supplementary Fig. S2).

### Both SPI-1^OFF^ and SPI-1^ON^ subpopulations are necessary for optimal invasion of epithelial cells

**Figure 3:**
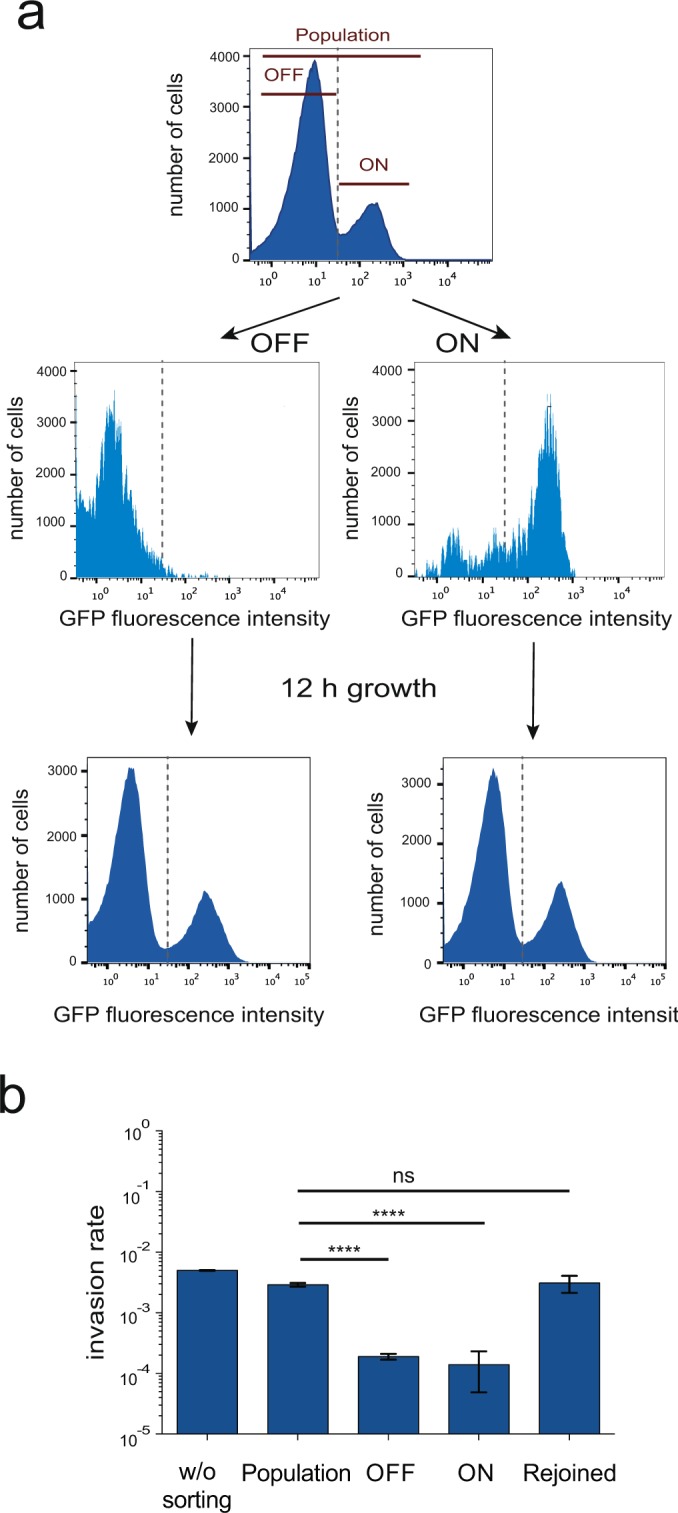
Invasion of epithelial cells by SPI-1^ON^ and SPI-1^OFF^ subpopulations. (**a**) (Upper) Flow cytometry analysis of GFP fluorescence intensity in strain SV7884 (*sipB*::GFP) before and after cell sorting. Two gates were drawn to separate cells that expressed *sipB::*GFP (SPI-1^ON^ subpopulation) from cells that did not (SPI-1^OFF^ subpopulation). After sorting, aliquots of sorted cells were run again at the cytometer to confirm the efficacy of sorting. (Lower) GFP fluorescence intensity of sorted SPI-1^ON^ and SPI-1^OFF^ subpopulations resuspended in LB broth and grown for 12 h under oxygen-limited conditions. (**b**) Invasion rates of sorted SPI-1^ON^ and SPI-1^OFF^ subpopulations. Invasion assays were performed using aliquots from the original culture (“w/o sorting”), from the whole bacterial population after passage through the cell sorter (“population”), from SPI-1^ON^ and SPI-1^OFF^ subpopulations, and from SPI-1^ON^ and SPI-1^OFF^ subpopulations mixed after sorting (“rejoined”) and incubated for 15 minutes before the invasion assay. Averages and standard deviations from >3 independent experiments are shown. Statistical indications: ns, not significantly different; **** significantly different, P < 0.0001.

As positive and negative controls for the experiments described in Fig. 1, we performed epithelial cell invasion assays using SPI-1^OFF^ and SPI-1^ON^ subpopulations separated by cell sorting (Fig. 3, upper panel a). As expected, the SPI-1^OFF^ subpopulation was less invasive than the unsorted population made of SPI-1^ON^ and SPI-1^OFF^ cells. However, an unsuspected observation was that the SPI-1^ON^ subpopulation was also less invasive. In the experiments summarized in Fig. 3, panel b, performed with subpopulations sorted under oxygen-limited conditions at late exponential growth (OD_600_ ~ 0.65), both the SPI-1^OFF^ and SPI-1^ON^ subpopulations showed invasion rates lower than an unsorted population. The paradox was confirmed when the sorted SPI-1^OFF^ and SPI-1^ON^ populations were mixed to re-construct a bistable population: the invasion rate of the re-constructed population was 23 and 16 fold higher than that of the sorted SPI-1^ON^ and SPI-1^OFF^ subpopulations, respectively, and similar to the invasion rate of the unsorted population (Fig. 3, panel b). Therefore, the surprising conclusion from these experiments was that both SPI-1^OFF^ and SPI-1^ON^ *Salmonella* cells are necessary for optimal invasion.

The observation that a pure SPI-1^ON^ population of *Salmonella* cells was less invasive than a mixture of SPI-1^ON^ and SPI-1^OFF^ cells was odd enough to consider the possibility that mutants might appear. However, the sorted SPI-1^OFF^ and SPI-1^ON^ subpopulations were able to re-generate a bistable population made of SPI-1^OFF^ and SPI-1^ON^ cells after growth during 12 h under invasive conditions (LB, oxygen limitation). Reversibility of the SPI-1^OFF^ and SPI-1^ON^ states thus ruled out the accumulation of mutants (Fig. 3, lower panel a). A slight decrease (about 2-fold) in the invasion rate was observed in the re-constructed population upon comparison with an unsorted population: 0.031 *vs* 0.050, respectively (shown also in Fig. 3, panel b). This observation might reflect the occurrence of minor cell damage upon cell sorting, but the effect was small enough to discard the possibility that sorting had irreversibly damaged the T3SS in the SPI-1^ON^ subpopulation.

### Single cell analysis of invasion of epithelial cells by SPI-1^OFF^ and SPI-1^ON^*Salmonellae*

**Figure 4:**
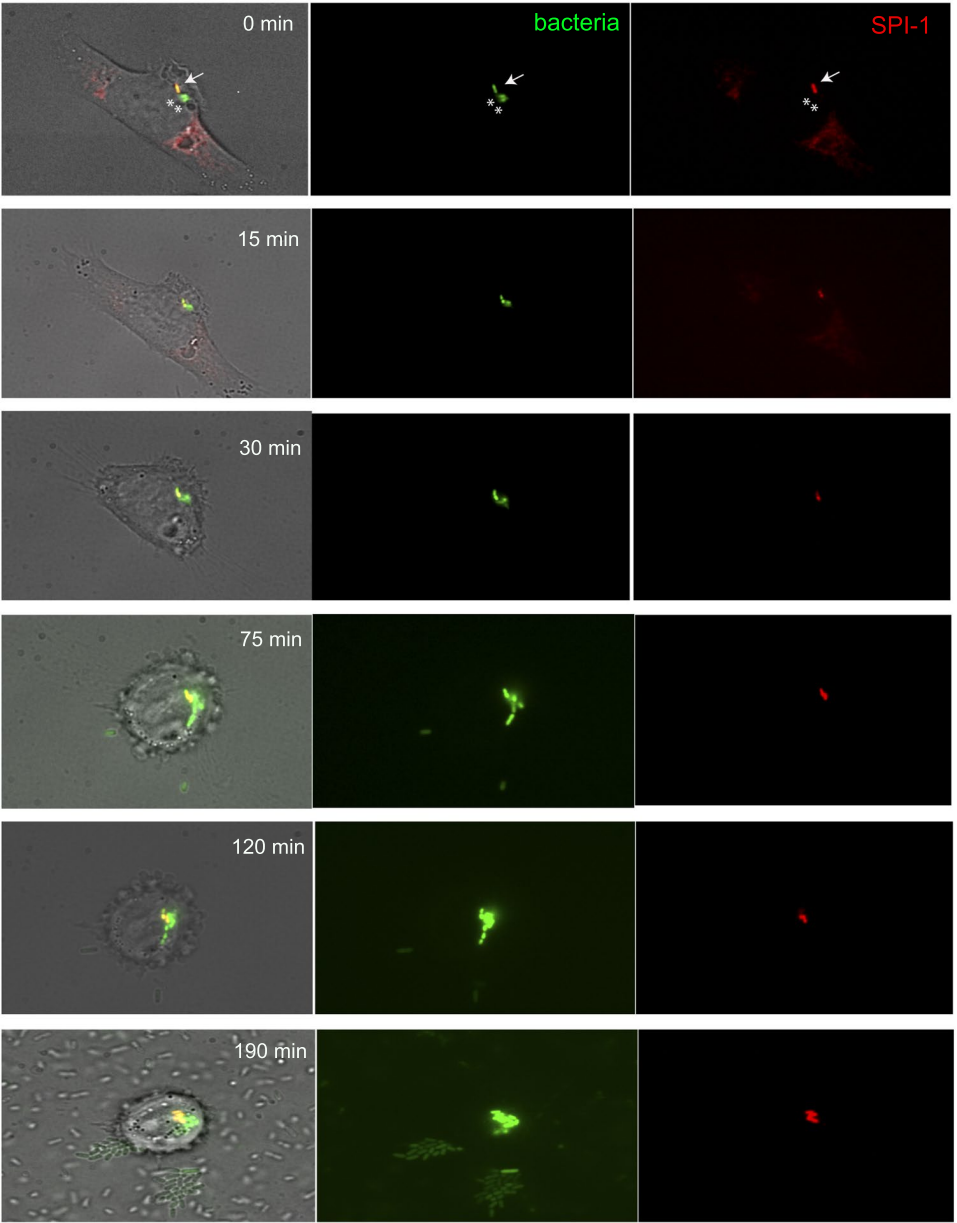
Visualization of *S. enterica* SV9250 *(sipB*::mCherry *ompC*::GFP) inside HeLa cells by fluorescence microscopy. In the central panel, all bacterial cells inside epithelial cells are green. In the right panel, bacterial cells that express SPI-1 are red. Images were taken periodically after infection, at the times indicated. In the top panel, arrows highlight cells that express SPI-1, whereas asterisks highlight *Salmonella* SPI-1 negative cells.

The observation that optimal invasion required the presence of both SPI-1^OFF^ and SPI-1^ON^ cells led us to investigate the types of bacterial cells found inside epithelial cells. The presence of SPI-1^OFF^ *Salmonella* inside epithelial cells was described before the advent of single cell analysis: an *S. enterica* mutant lacking the SPI-1 protein InvE was able to invade epithelial cells in the presence of the wild type^42^. Single cell analysis of invasion by SPI-1^OFF^ and SPI-1^ON^ cells was performed using a strain that carried a mCherry transcriptional fusion downstream of *sipB* (*sipB*::mCherry) and a GFP transcriptional fusion downstream of *ompC* (SV9250). Choice of mCherry was based on its higher stability and slower photobleaching compared with other fluorescent proteins^44^. HeLa cells were infected and washed to eliminate extracellular bacterial cells. When individual *Salmonellae* were visualized inside epithelial cells by fluorescence microscopy, the intracellular bacterial populations were found to be made of both SPI-1^ON^ and SPI-1^OFF^ cells (Fig. 4). The possibility that intracellular SPI-1^OFF^ cells were actually SPI-1^ON^ cells that had switched off SPI-1 seemed unlikely given the photostability of the fluorescent protein used. The opposite artefact (that some SPI-1^ON^ cells might remain fluorescent after turning off SPI-1) was thus more likely, perhaps underestimating the number of intracellular SPI-1^OFF^ cells. Hence, single cell analysis confirms that T3SS-mediated secretion by the SPI-1^ON^ subpopulation is not a *sine qua non* condition for invasion, and that “rescue” can occur^42^. A tentative interpretation is that ruffling and other bacterial manipulations of the host cell may allow SPI-1^OFF^ *Salmonellae* to enter epithelial cells.

### Discrimination of *Salmonella* during epithelial cell invasion

**Figure 5:**
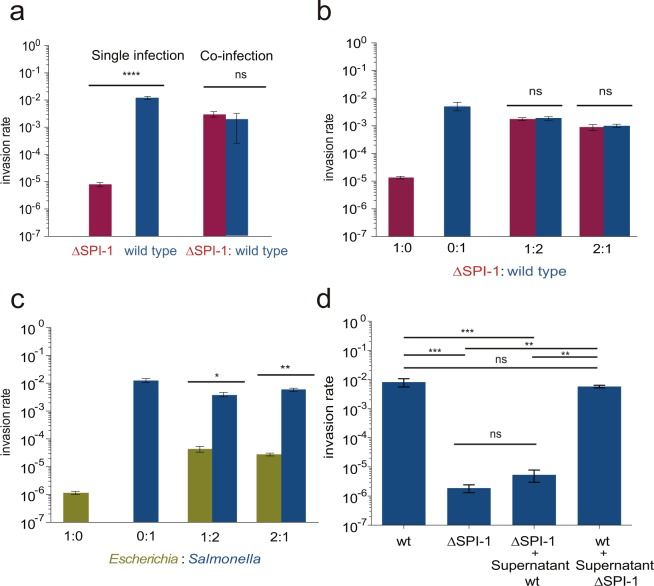
Invasion of epithelial cells by strains possessing or lacking SPI-1. (**a**) Comparative analysis of invasion of epithelial cells by *Salmonella enterica* strain SV9244 (ΔSPI-1) upon single infection and upon mixed infection with SV7884 (*sipB::*GFP) in a 1:1 proportion. Blue bars: SV7224; SV9244: red bars. (**b**) Invasion of epithelial cells by *Salmonella enterica* strain SV9244 (ΔSPI-1) upon single infection and upon mixed infection with SV7884 (*sipB::*GFP) in various proportions. (**c**) Invasion rates of *S. enterica* upon mixed infections with *Escherichia coli* (green bars) using different *Escherichia*:*Salmonella* ratios. (**d**) Invasion of epithelial cells by the wild type (wt), SV9244, a strain lacking SPI-1 (ΔSPI-1), a culture made from cells originated during ΔSPI-1 culture and supernatant from a wild type culture, and a culture made from cells generated by a wild type culture and supernatant from ΔSPI-1 culture. In all experiments, antibiotic markers were used to distinguish the strains. Averages and standard deviations from >3 independent experiments are shown. Statistical indications: ns, not significantly different; ****P < 0.0001, ***P < 0.001, **P < 0.01, *P < 0.05, Student’s t-test.

To investigate whether invasion of epithelial cells by SPI-1^OFF^ *Salmonellae* was species-specific, we compared the invasion capacity of an *S. enterica* strain lacking SPI-1 with the invasion capacity of an *Escherichia coli* strain. For this purpose, we infected HeLa epithelial cells with a mixed population made of the wild type (which undergoes bistable SPI-1 expression) and of SV9244 (ΔSPI-1). The mixture was prepared in a 1:1 proportion. As controls, infections were also performed with the wild type and the ΔSPI-1 strain. Bacterial cells recovered after epithelial cell invasion were plated on LB and LB supplemented with appropriate antibiotics (kanamycin or chloramphenicol), and invasion rates were calculated from the numbers of colonies formed by each strain. The invasion rate of the ΔSPI-1 strain was similar to that of the wild type upon mixed infection (Fig. 5, panel a), and more than two orders of magnitude higher than the invasion rate of the ΔSPI-1 strain alone. Congruent observations were made when the wild type and the ΔSPI-1 strain were mixed in 1:2 and 2:1 proportions (Fig. 5, panel b). In contrast, *E. coli* cells mixed with wild type *Salmonellae* at different proportions did not invade epithelial cells at significant levels (Fig. 5, panel c). Discrimination indicates that rescue of SPI-1^OFF^ cells by SPI-1^ON^ cells is a *Salmonella*-specific trait. Recruitment of *Salmonella* cells at specific epithelium sites may play a role in species discrimination^45^.

To test whether rescue involved diffusible factors, we added the supernatant fraction of an invasive culture to a pellet of cells lacking SPI-1, and performed invasion assays in the customary way. The results were unambiguous: the supernatant from the wild type grown under invasion conditions did not confer invasiveness to ΔSPI-1 *Salmonella* cells, suggesting that an extracellular diffusible factor is not involved (Fig. 5, panel d).

### A small number of SPI-1^ON^ cells is sufficient for invasion

**Figure 6:**
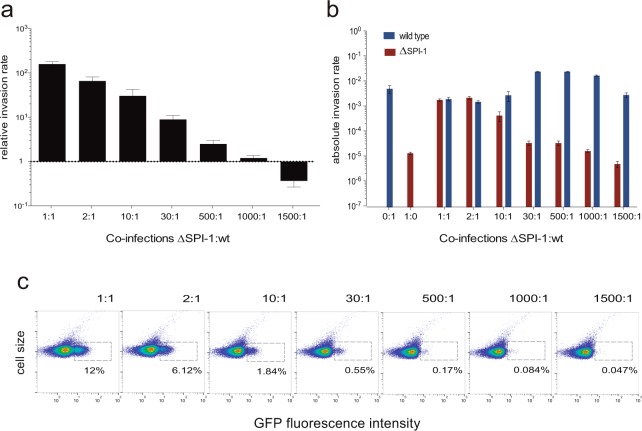
Correlation between the invasion rate of epithelial cells and the number of SPI-1^ON^ cells. (**a**) Relative invasion rates of epithelial cells by different proportions of ΔSPI-1 and wild type cells. Invasion rates were normalized to the invasion rate of the ΔSPI-1 strain in a single infection. Note that bars/values below 1 indicate that a strain lacking SPI-1 (SV9244) grown in a mixed culture invades epithelial cells better than in a single infection. (**b**) Absolute invasion rates of epithelial cells by ΔSPI-1 (red bars) and wild type (blue bars) cells mixed at different proportions. (**c**) GFP fluorescence distribution in mixed cultures made of ΔSPI-1 and wild type cells at different proportions. The fraction of cells expressing SPI-1 in each culture is indicated.

Because higher proportions of SPI-1^ON^ cells do not increase the rate of epithelial cell invasion and SPI-1^OFF^ cells are able to invade upon co-infection with SPI-1^ON^ cells, we wondered whether a threshold proportion of SPI-1^ON^ cells might be required to foster invasion. To address this issue, we prepared bacterial suspensions containing different proportions of SPI-1^ON^ and SPI-1^OFF^ cells. Mixtures were prepared from cultures of the wild type and a SPI-1 lacking strain. Invasion assays were performed using a constant multiplicity of infection. Discrimination between the two strains was based, as above, on use of antibiotic resistance markers. The absolute and relative invasion rates of the ΔSPI-1 strain mixed with the wild type at different proportions are shown in Fig. 6, panels a and b. In turn, the fraction of cells expressing SPI-1 at each proportion is represented in Fig. 6, panel c. The term “relative invasion ratio” indicates that the numbers are relative to the invasion rate of the ΔSPI-1 strain alone, arbitrarily adjusted to “1”. A small subpopulation of SPI-1^ON^ cells (around 0.1%, Fig. 6 panel c) was able to trigger invasion by cells lacking the invasion machinery (in other words, unable to invade on their own). Hence, the distribution of labour between SPI-1^ON^ and SPI-1^Off^ cells can be skewed without endangering the invasion capacity of the whole *Salmonella* population.

## Discussion

Bacterial adaptation to multiple environments is made possible by signal transduction systems, hierarchical gene networks, stress responses, DNA repair systems, efflux pumps, and other devices responsive to environmental signals. However, the adaptation ability is limited by the fact that all organisms have a limited set of possible traits, and a given phenotype can only be improved at the expense of another phenotype^41^. Hence, adaptation necessarily requires tradeoffs^13,46^.

Formation of bacterial subpopulations can provide a solution to the tradeoff problem by allotting to two or more bacterial lineages the benefits and payoffs associated to specific phenotypes^6^. In bacterial pathogens, programmed heterogeneity can help to evade the immune system and other host defenses, and can adapt bacterial subpopulations to distinct host environments^5,47^. In many cases, however, the payoffs associated to bistability remain unknown. A remarkable exception is bistable expression of SPI-1 in *S. enterica* serovar Typhimurium. Indeed, Wolf-Dietrich Hardt and co-workers have examined the benefits and payoffs of SPI-1 bistability upon colonization of animal hosts, and have pondered the adaptive value of subpopulation formation. The SPI-1^ON^ subpopulation builds the T3SS apparatus that permits invasion of epithelial cells at the expense of slow growth^30^; however this subpopulation is antibiotic-resistant^38^. In turn, the SPI-1^OFF^ subpopulation does not build the invasion T3SS but benefits from the inflammatory response triggered by the SPI-1^ON^ subpopulation^37,39^. Formation of a large SPI^OFF^ subpopulation may be crucial for intestinal persistence, avoiding takeover of the *Salmonella* population by fast-growing avirulent mutants^39,40^. Altogether, the combination of benefits and payoffs associated to SPI-1 bistability fits in a strategy, named “cooperative virulence” by Hardt and co-workers, that contributes to preservation of virulence in the *Salmonella* population. This strategy may warrant shedding of a virulent population to the environment and subsequent transmission to new hosts.

This study has analyzed epithelial cell invasion by *Salmonella enterica* ser. Typhimurium using single cell techniques that permit detection of bacterial subpopulations, assessment of their sizes, and separation by cell sorting. The observations made with this approach have provided unsuspected observations that add refinements to the current knowledge of SPI-1 bistability. For instance, contrary to intuition, the invasion rate of a *Salmonella* population varies very little with different proportions of SPI-1^OFF^ and SPI-1^ON^ cells (Fig. 1). In fact, a low number of SPI-1^ON^ cells is sufficient to confer invasiveness to the *Salmonella* population: epithelial cell invasion is still observed with a proportion as low as 0.17% SPI-1^ON^ cells (Fig. 5). This trait is potentially relevant for host colonization given the bottlenecks encountered by *Salmonella* populations inside animals^48^ and the slow growth of SPI-1^ON^ cells^30^. Flexible division of labour may thus confer robustness of the system: invasion will occur regardless of the sizes of the SPI-1^ON^ and SPI-1^OFF^ subpopulations.

Another unsuspected finding of this study is that optimal invasion of epithelial cells requires the presence of both SPI-1^ON^ and SPI-1^OFF^ cells (Fig. 2). Increased invasion rates are also detected upon re-construction of a population containing SPI-1^ON^ and SPI-1^OFF^ cells (Fig. 2). The observation that a mixed population is better suited for invasion than a SPI-1^ON^ subpopulation alone is paradoxical, and supports the unorthodox conclusion that the SPI-1^OFF^ subpopulation has an active role in invasion. Because SPI-1^OFF^ cells grow faster^30^, they can expected to be the most abundant cell type, and their ability to invade may optimize the invasion process. A more risky speculation is that fast growth rate may be accompanied by other SPI-1^OFF^-specific traits that facilitate invasion. Epithelium invasion by SPI-1^OFF^ cells may extend cooperative virulence to the intracellular environment by competing with mutants able to invade the epithelium. Invasion by SPI-1 mutants was described in the 1990′s by Jorge Galán and co-workers^42^, and has been reproduced in this study using single cell analysis (Fig. 4). The existence of species discrimination for epithelial cell invasion (Fig. 4) further strengthens the view that invasion by SPI-1^OFF^ cells may be part of an evolutionary strategy for preservation of *Salmonella* virulence.

## Methods

### Bacterial strains

Strains of *Salmonella enterica* serovar Typhimurium used in this study derive from the mouse-virulent strain SL1344^49^. For simplicity, *Salmonella enterica* serovar Typhimurium is abbreviated as *S. enterica* in the text. Strains constructed for this study are listed in Table S1. Strain SV6811 (*ompC*::GFP (Cm^R^)) was described elsewhere^50^. Strain SV9244 (∆SPI-1) was constructed by disruption and replacement of a 34.8 kb region within SPI-1 with a Km resistance cassette. Transcriptional fusions with the *gfp* and *mCherry* genes were constructed downstream of the stop codon of *sipB*, generating strains SV7884 and SV8302, respectively. The source of the promoterless *GFP* gene and the chloramphenicol resistance cassette was pZEP07^29^. For the construction of strain SV8302 (*sipB*::mCherry), a DNA fragment containing the promoterless *mCherry* gene and the kanamycin resistance cassette was PCR-amplified from pDOC-R, an *mCherry*-containing derivative of plasmid pDOC (a gift from Steve Busby’s lab). Addition of a 3xFLAG epitope tag to protein-coding DNA sequences was carried out using plasmid pSUB11 (Km^r^, 3xFLAG) as template^51^ to generate SV9418 (*sipB*::3xFLAG). PCR products used for construction of strains SV9244 (∆SPI-1), SV7884 (*sipB*::GFP), SV8302 (*sipB*::mCherry), and SV9418 (*sipB*::3xFLAG) were integrated into the chromosome of SL1344 using the Lambda Red recombination system^52^. All primers used in strain construction are shown in Table S2. P22 HT-mediated transduction was used to generate strain SV9250 (*ompC*::GFP *sipB*::mCherry) using SV6811 (*ompC*::GFP) as donor and SV8302 (*sipB*::mCherry) as recipient. *E. coli* pDOC-G (Km^R^) is a DH5α derivative carrying plasmid pDOC-G (Km^R^)^53^.

### Media and culture conditions

Bertani’s lysogeny broth (LB) was used as liquid medium. Cultures were grown at 37 °C without shaking in borosilicate tubes with 5 ml of growth medium (oxygen-limited, “invasive” condition). When unimodal SPI-1 expression was required, the cultures were grown overnight with shaking for 18 hours at 37 °C (non-invasive condition).

### Invasion assay in HeLa epithelial cells

HeLa human epithelial cells (ATCC CCL-2) were grown in DMEM containing 10% fetal calf serum and 1 mM glutamine (Life Technologies). HeLa cells were seeded the day before the infection in 24-well plates (Costar, Corning). Cells were grown and prepared in DMEM. The colony forming units (CFU) of the one or two strains in the input were enumerated by plating a dilution series of the inoculum, using the appropriate antibiotic to distinguish the strains. The bacterial mixture was added to HeLa cells to reach a multiplicity of infection (MOI) of 75 bacteria per eukaryotic cell. 30 minutes after the infection, cells were washed twice with phosphate buffered saline (PBS) and incubated in fresh DMEM medium containing 100 μg/ml gentamicin for 90 minutes. Numbers of viable intracellular bacteria were obtained after lysis of infected cells with 1% Triton X-100, and plating on appropriate media. Infections were carried out in triplicate. The invasion rate is defined as the ratio between the output (intracellular bacteria recovered 2 h after infection) and the input (initial inoculum). Mixed bacterial suspensions containing different proportions of SPI-1^OFF^ and SPI-1^ON^ cells were prepared from cultures of SV9244 (ΔSPI-1) and SV7884 (wild type, *sipB*::*GFP*). The mixtures were incubated for 15 min before the invasion assay.

### Invasion assay using pellets of cells and supernatant

To obtain the supernatant fraction, the cultures were centrifuged for 5 min at 13,000 × rpm and the supernatant of each sample was collected and filtered through a low protein binding 0.22-μm-pore-size filter to remove residual bacterial cells. To obtain the pellet of cells, cultures were centrifuged for 5 min at 13,000 × rpm, the sedimented cells were washed with 1 ml of PBS, and re-suspended in different supernatants. Subsequently, invasion assays were performed as described previously.

### Statistical analysis

One-way ANOVA and Student’s t-test (two-tailed) were performed using GraphPad Prism version 6.0 for Mac. The one-way ANOVA test was used to determine whether differences in invasion rates along the cell cycle were statistically significant (p < 0.05). The Student’s *t* test was used to determine statistical differences between two groups.

### Flow cytometry analysis of SPI-1 expression

Flow cytometry was used to monitor SPI-1 expression using a *sipB*::GFP, *hilA*::GFP, and *prgH*::GFP fusions. Data acquisition was performed using a Cytomics FC500-MPL cytometer (Beckman Coulter, Brea, CA) and data was analyzed with FlowJo X version 10.0.7r software (Tree Star, Inc.). *S. enterica* strains were grown at 37 °C until desired optical density, washed, and re-suspended in phosphate-buffered saline (PBS) for fluorescence measurement by flow cytometry. Fluorescence values for 100.000 events were compared with the data from the reporterless control strain, thus yielding the fraction of SPI-1^ON^ cells.

### Fluorescence activated cell sorting (FACS) of live cells

Cells from an overnight culture were grown under invasive conditions. The culture was washed and re-suspended in PBS to a final concentration of 5 × 10^6^ cells/ml. Cells were sorted using a MoFlo Astrios EQ cytometer (Beckman Coulter, Brea, CA). Immediately prior to sorting, cells were analyzed for GFP expression. Based on this analysis, gates were drawn to separate cells that expressed GFP (SPI-1^ON^ state) from cells that did not express GFP levels (SPI-1^OFF^ state). From each gate, cells were collected into a sterile tube. After sorting, cells were spun at 4,000 rpm for 10 min. FACS buffer was then removed, and cells were re-suspended in DMEM to perform invasion assays. An aliquot of sorted cells was run again at the cytometer to confirm the purity of the preparation. Data were obtained with Summit v6.2 and analyzed with FlowJo X version 10.0.7r software (Tree Star, Inc.).

### Fluorescence microscopy

Strain SV9250 (*ompC*::*GFP* *sipB*::*mCherry*) was grown in LB medium without shaking for 18 hours at 37 °C. An appropriate proportion of bacterial cells was prepared in DMEM and added to HeLa cells to reach a MOI of 75 bacteria per eukaryotic cell. Thirty minutes after infection, cells were washed twice with PBS to eliminate extracellular bacterial cells, and resuspended in DMEM medium. Images were captured with a Zeiss Apotome fluorescence microscope equipped with a 100x Plan Apochromat objective and an incubation system that covers every requirement in the cultivation and observation of living cells (37 °C; 5% CO_2_). Pictures were taken at different times using an Axiocam 506 camera, and the images were analyzed using ImageJ software (Wayne Rasband, Research Services Branch, National Institute of Mental Health, MD, USA).

### Determination of SPI-1^ON^ cells by immunostaining

The number of SPI-1^ON^ cells was measured by immunostaining, as described previously^54^, treating ethanol-fixed cells of strain SV9418 (*sipB*::3xFLAG) with anti-FLAG® M2 monoclonal antibody (Sigma-Aldrich) and a secondary antibody conjugated to Alexa Fluor® 488 (Life Technologies). Immunostaining was followed by flow cytometry using a Cytomics FC500-MPL cytometer (Beckman Coulter, Brea, CA). Data were obtained with CXP software (Beckman Coulter, Brea, CA) and analyzed with FlowJo X version 10.0.7r software (Tree Star, Inc.).

## Electronic supplementary material

Supplementary material
