# Supplementary material for: Contribution of SPI-1 bistability to Salmonella enterica cooperative virulence: insights from single cell analysis
Source: Sci Rep. 2018 Oct 5;8:14875. doi: 10.1038/s41598-018-33137-z (PMC6173691; doi:10.1038/s41598-018-33137-z)
Supplement: Supplementary file 1 — Supplementary material [file 41598_2018_33137_MOESM1_ESM.pdf]

Contribution of SPI-1 bistability to *Salmonella enterica* cooperative virulence: insights from single cell analysis

María Antonia Sánchez-Romero and Josep Casadesús

SUPPLEMENTARY MATERIAL

Table S1. *S. enterica* strains constructed for this study

Strain	Genotype
SV7884	SL1344 *sipB*::GFP (Cm^R)
SV8302	SL1344 *sipB*::mCherry (Km^R)
SV9244	SL1344 ΔSPI-1::Km^R
SV9250	SL1344 *sipB*::mCherry (Km^R) *ompC*::GFP (Cm^R)
SV9418	SL1344 *sipB*::3xFLAG(Km^R)
SV8348	SL1344 *prgH::*GFP (Cm^R) inserted at the *putPA* locus*
SV6806	SL1344 *hilA*::GFP (Cm^R)

* Constructed by P22 HT-mediated transduction using JH3010[1] as a donor.

1. Hautefort, I., Proença, M. J. & Hinton, J. C. D. Single-copy green fluorescent protein gene fusions allow accurate measurement of Salmonella gene expression *in vitro* and during infection of mammalian cells. *Appl. Environ. Microbiol.* **69,** 7480–7491 (2003).

Table S2. Oligonucleotides used in this study

Oligonucleotide name	Sequence (5'-3')
**SPI1P1**	AGCATAACGGCATTGTTATCGAATCGCTCATAAAGCGTTTGTGTAG GGAGCTGCTTC
**SPI1P2**	TATAAGGCTTGCAGTCTTTCATGGGCAGCAAGTAACGTCTCATATG TCCTCCTTAG
**sipB-GFP-FOR**	AGCAAAATGCGGATGCTTCGCGTTTTATTCTGCGCCAGAGTCGCG ATAAGAAGGAGATATACATATGAG
**sipB-GFP-REV**	ATTCCCACATTACTAATTAACATATTTTTCTCCCTTTATTTTGGCAGT ATCACTTATTCAGGCGTA
**sipB-mch-F**	AGCAAAATGCGGATGCTTCGCGTTTTATTCTGCGCCAGAGTCGCG ATAAGAAGGAGTATACATATGGTGAGCAAGGGC
**sipB-mCh-R**	ATTCCCACATTACTAATTAACATATTTTTCTCCCTTTATTTTGGCAGT AATATCCTCCTTAGTTCC
**P1-sipB-F**	AGCAAAATGCGGATGCTTCGCGTTTTATTCTGCGCCAGAGTCGCG CTACAAAGACCATGACGG
**P2-sipB-R**	ATTCCCACATTACTAATTAACATATTTTTCTCCCTTTATTTTGGCAGT TATGAATATCCTCCTTAG
**hilA-GFP-FOR**	TTTGGTTCAAAAGATGGAAACAGGATCCCCGCTTGATTAAATTACG TAAGAAGGAGATATACATATGAG
**hilA-GFP-FOR**	CAACCAGATTACGATGATAAAAAAATAATGCATATCTCCTCTCTCA[illegible] TATCACTTATTCAGGCGTA

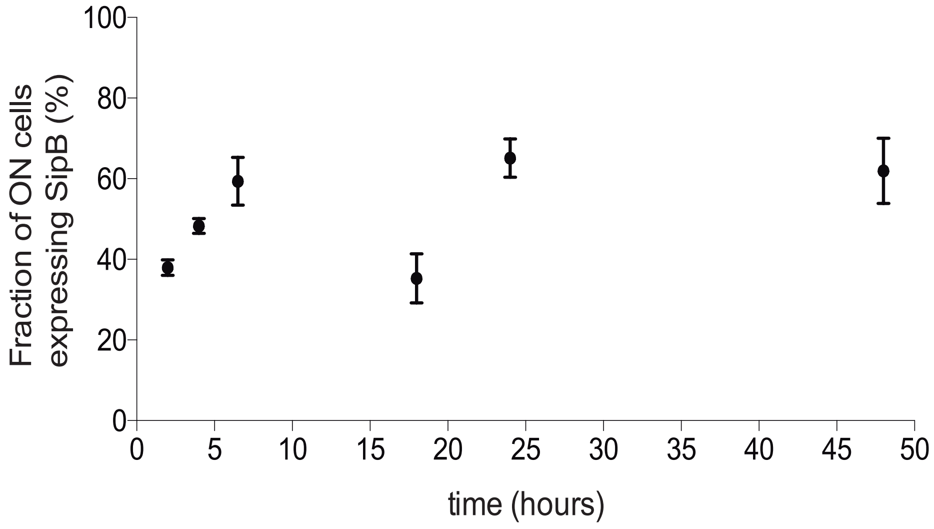

Figure S1. Fractions of SPI-1^{ON} *Salmonella* cells at different stages of the cell cycle analyzed by immunostaining. The strain used was SV9418 (*sipB::*3xFLAG).

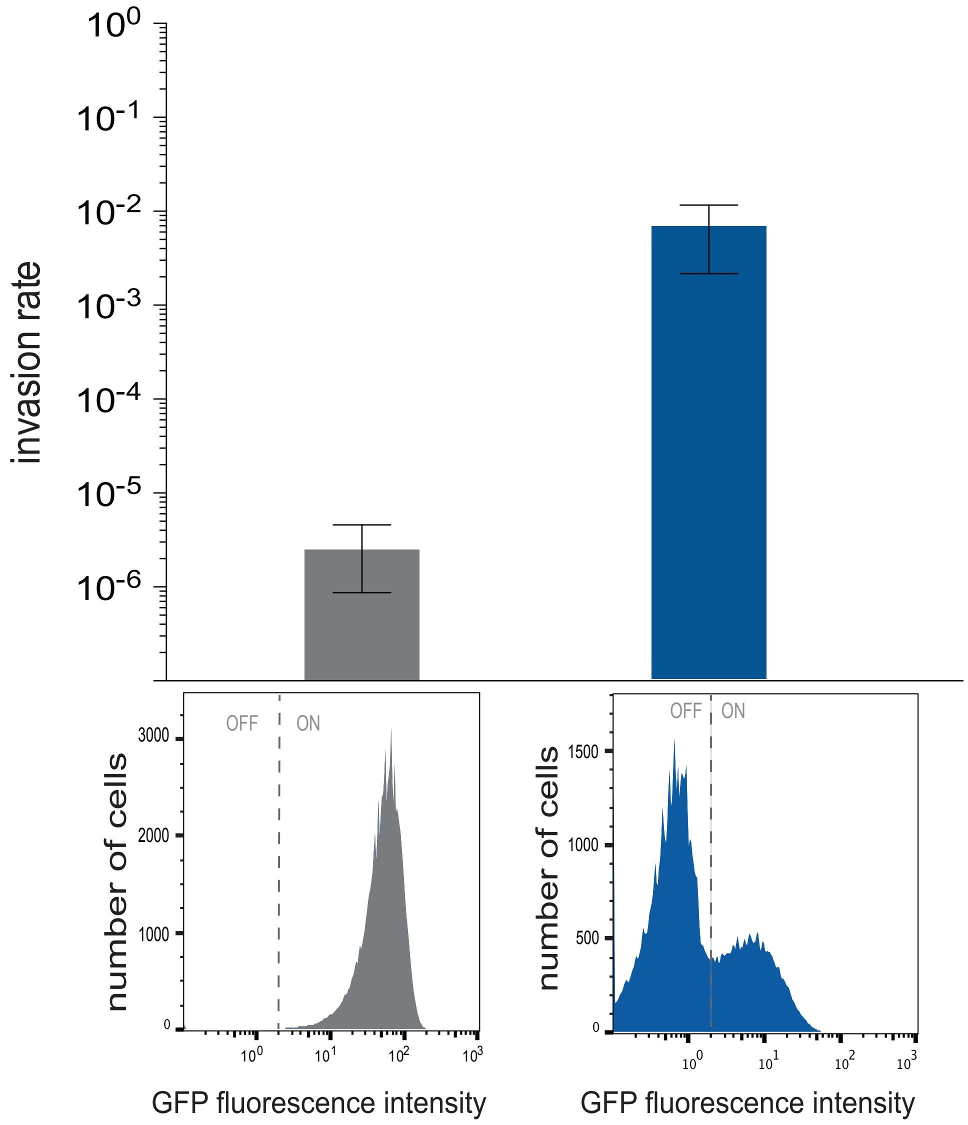

Figure S2. Invasion of epithelial cells by strain SV7884 (*sipB*::GFP*)* grown under growth conditions that permit unimodal or bimodal expression of SPI-1. Expression of *sipB::*GFP and invasion of epithelial cells were analyzed under aeration (grey bar and histogram) and under oxygen limitation (blue bar and histogram).
